# Biomarkers associated with delirium in critically ill patients and their relation with long-term subjective cognitive dysfunction; indications for different pathways governing delirium in inflamed and noninflamed patients

**DOI:** 10.1186/cc10598

**Published:** 2011-12-29

**Authors:** Mark van den Boogaard, Matthijs Kox, Kieran L Quinn, Theo van Achterberg, Johannes G van der Hoeven, Lisette Schoonhoven, Peter Pickkers

**Affiliations:** 1 Department of Intensive Care Medicine, Radboud University Nijmegen Medical Centre, P.O. Box 9101, Nijmegen, 6500HB, the Netherlands; 2 Departments of Anesthesia and Critical Care, St. Michael's Hospital, 30 Bond St., Toronto, ON M5B 1W8, Canada; 3 Nijmegen Institute for Infection, Inflammation and Immunity (N4i), Radboud University Nijmegen, Medical Centre, PO Box 9101, 6500 HB, Nijmegen, the Netherlands; 4 Scientific Institute for Quality of Healthcare, Radboud University Nijmegen Medical Centre, PO Box 9101, 6500 HB, Nijmegen, the Netherlands

## Abstract

**Introduction:**

Delirium occurs frequently in critically ill patients and is associated with disease severity and infection. Although several pathways for delirium have been described, biomarkers associated with delirium in intensive care unit (ICU) patients is not well studied. We examined plasma biomarkers in delirious and nondelirious patients and the role of these biomarkers on long-term cognitive function.

**Methods:**

In an exploratory observational study, we included 100 ICU patients with or without delirium and with ("inflamed") and without ("noninflamed") infection/systemic inflammatory response syndrome (SIRS). Delirium was diagnosed by using the confusion-assessment method-ICU (CAM-ICU). Within 24 hours after the onset of delirium, blood was obtained for biomarker analysis. No differences in patient characteristics were found between delirious and nondelirious patients. To determine associations between biomarkers and delirium, univariate and multivariate logistic regression analyses were performed. Eighteen months after ICU discharge, a cognitive-failure questionnaire was distributed to the ICU survivors.

**Results:**

In total, 50 delirious and 50 nondelirious patients were included. We found that IL-8, MCP-1, procalcitonin (PCT), cortisol, and S100-*β *were significantly associated with delirium in inflamed patients (*n *= 46). In the noninflamed group of patients (*n *= 54), IL-8, IL-1ra, IL-10 ratio A*β*_1-42/40_, and ratio A*β*_N-42/40 _were significantly associated with delirium. In multivariate regression analysis, IL-8 was independently associated (odds ratio, 9.0; 95% confidence interval (CI), 1.8 to 44.0) with delirium in inflamed patients and IL-10 (OR 2.6; 95% CI 1.1 to 5.9), and A*β*_1-42/40 _(OR, 0.03; 95% CI, 0.002 to 0.50) with delirium in noninflamed patients. Furthermore, levels of several amyloid-*β *forms, but not human Tau or S100-*β*, were significantly correlated with self-reported cognitive impairment 18 months after ICU discharge, whereas inflammatory markers were not correlated to impaired long-term cognitive function.

**Conclusions:**

In inflamed patients, the proinflammatory cytokine IL-8 was associated with delirium, whereas in noninflamed patients, antiinflammatory cytokine IL-10 and A*β*_1*-*42/40 _were associated with delirium. This suggests that the underlying mechanism governing the development of delirium in inflamed patients differs from that in noninflamed patients. Finally, elevated levels of amyloid-*β *correlated with long-term subjective cognitive-impairment delirium may represent the first sign of a (subclinical) dementia process. Future studies must confirm these results.

The study was registered in the Clinical Trial Register (NCT00604773).

## Introduction

Delirium is a serious and frequently occurring disorder in critically ill patients associated with both physical and cognitive impaired outcome [1-4]. Because the pathogenesis of delirium is probably multifactorial, biomarker analysis may provide valuable information regarding the underlying mechanisms [5-7].

Several previous investigations in non-ICU patients established an association between inflammation and delirium, as correlations between proinflammatory cytokine levels and delirium have been found [6,8-10]. Furthermore, in elderly delirious patients with hip fractures, increased concentrations of IL-6, IL-8, and cortisol were correlated with elevated levels of the brain-specific protein (BSP) S100-*β *(a marker for astrocyte damage) [11]. Interestingly, sepsis is also associated with elevated levels of BSP [12,13]. Furthermore, it has been hypothesized that serious illness such as sepsis, as well as the use of sedatives and analgesic, could result in apoptosis and long-term cognitive impairment [14]. In mice, tumor-necrosis factor (TNF)-α is a mediator of apoptotic cellular death in the brain [15] and may therefore be causally associated with the development of delirium in patients with severe inflammation.

In the long term, delirium is associated with a more than 12-fold increased risk for developing dementia [16], resulting in permanent impairment of cognitive function that is associated with altered levels of amyloid-*β *[17,18]. The association between biomarkers in delirious patients and long-term cognitive function is unknown.

With regard to its multifactorial nature, it is likely that the underlying mechanisms of delirium may differ between inflamed and noninflamed patients. In the present study, we explored which biomarkers were associated with delirium in inflamed patients and which were associated with delirium in noninflamed patients, thereby using these biomarkers to explore whether different underlying mechanisms are involved. We included biomarkers that are directly linked to delirium, as determined in previous studies, and biomarkers that are linked with the onset of delirium. Apart from well-established pro- and antiinflammatory cytokines, we determined, for example, procalcitonin [19], macrophage migration inhibitory factor [20], and human neutrophil peptide-1 [21], which play a role in inflammation, directly associated with delirium [22]. Finally, we searched for correlations between mediators that were related to delirium and brain-specific proteins and cognitive functions 18 months after ICU discharge to establish whether the different pathways exert different long-term cognitive effects.

## Material and methods

### Patients and definitions

A convenience sample was taken of all medical and surgical patients older than 18 years admitted to our Intensive Care Department (tertiary referral hospital in Nijmegen, the Netherlands) between February and July 2008. These patients were screened for delirium by using the confusion-assessment method-ICU (CAM-ICU) [23,24]. Patients were excluded when delirium screening during patients' complete ICU stay could not be performed (for example, because of persistent coma). Patients who were admitted to the ICU for trauma, postcardiac arrest, or neurologic reasons were also excluded. Finally, patients were excluded when they had a history of serious cognitive impairment, defined as reported in their medical history, or had from any form of dementia, delirium, or obvious signs of cognitive impairment reported by their relatives. If doubt existed concerning preexistent cognitive function, patients were not included in this study.

In delirious patients, blood was drawn within 24 hours after the onset of delirium. For the nondelirious group, because no point in time exists to relate to, we draw blood after a similar ICU length of stay compared with that of the group of delirious patients. A total of 5 ml blood was drawn for all measurements.

Delirium and nondelirium patients were furthermore divided into inflamed and noninflamed patients. This distinction was made because inflamed patients are suspected to have high levels of inflammatory mediators, and from a group of noninflamed patients, it is expected that they have low levels of inflammatory mediators. Inflamed was defined as a positive culture, regardless the origin from which specimens were taken, for which the patient was treated with antibiotics. Although systemic inflammatory response syndrome (SIRS) criteria lack specificity, the study was designed to differentiate between inflamed patients and noninflamed patients, and therefore, we used the presence of more than two SIRS criteria as a marker of inflammation [25]. Absence of inflammation was defined as the absence of proven or suspected infection and the presence of no more than one SIRS criterion.

The regional Medical Ethics Committee of Arnhem-Nijmegen approved the study and waived the need for informed consent because a single blood withdrawal is not considered a burden for the patient, and the results of this study did not influence the standard care for that patient. The study was registered in the Clinical Trial Register (NCT00604773).

### Procedures

Demographic variables as well as illness-related characteristics were collected. The validated CAM-ICU method was used to detect whether patients were delirious. All patients were screened at least 3 times per day by using the CAM-ICU, with a high interrater reliability of 0.90 (95% CI, 0.82 to 0.98) Cohen kappa by well-trained ICU nurses [26]. Patients were diagnosed with delirium when they had at least one positive CAM-ICU screening. Patients without any positive CAM-ICU screening during the complete ICU stay were classified as nondelirious. In case of doubt regarding the delirium diagnosis, patients were not included in this study.

Blood for the determination of biomarkers was drawn between 6 and 10 a.m. and within 24 hours of the first positive CAM-ICU screening from an indwelling arterial line. Blood was centrifuged at 2,000 *g *for 15 minutes, and plasma was stored at -80°C until analysis. Proinflammatory cytokines (tumor necrosis factor (TNF)-α, interleukin (IL)-1*β*, IL-6, IL-8, IL-17, IL-18, and macrophage migration inhibitory factor (MIF), antiinflammatory cytokines (IL-1RA and IL-10), and the chemotactic cytokine MCP-1 were determined by using a simultaneous Luminex assay (Milliplex; Millipore, Billerica, MA, USA). Plasma defensin (human neutrophil peptide-1 (HNP-1)) was measured by using mouse anti-human HNP-1-3 monoclonal antibody (HyCult Biotechnology, Uden, The Netherlands), and the wells were then incubated with rabbit anti-human HNP-1-3 polyclonal antibody (Host Defence Research Centre, Toronto, ON, Canada), followed by incubation with peroxidase-conjugated goat-anti-rabbit IgG (Jackson ImmunoResearch). C-reactive protein (CRP) concentrations were measured by using immunologic detection (turbidimetric method, Aeroset; Abbott Laboratories, Abbott Park, IL, USA), and procalcitonin (PCT) levels were determined by using an immunometric assay with time-resolved amplified cryptate emission technology (PCT-sensitive Kryptor kit; Brahms, Middletown, VA, USA). The stress hormone cortisol was measured with luminometric immunoassay on a random-access analyzer (Architect *i *System, Abbott).

The brain-specific proteins full-length amyloid*β*_1-42 _and _1-40 _(A*β*_1-42 _and A*β*_1-40_) and truncated A*β*-42 and -40 (A*β*_N-42 _and A*β*_N-40_) were determined in plasma by using a simultaneous Luminex assay (INNO-BIA plasma A*β *forms; Innogenetics, Ghent, Belgium), which has been shown to be a reliable assay with a low variability [27]. Plasma levels of S100 calcium-binding protein-*β *(S100-*β) *and total human Tau were analyzed by using two commercially available enzyme-linked immunosorbent assay (ELISA) kits (Cosmo Bio Co., Ltd., Tokyo, Japan; and Cusabio Biotech Co. Ltd., Donghu; China, respectively). All biomarkers were determined according to the manufacturer's instructions.

### Subjective long-term cognitive functioning

All included patients received the validated Dutch translation [28] of the cognitive-failure questionnaire (CFQ) 18 months (median) after discharge from the ICU [29]. The self reported CFQ measures four dimensions of cognition: memory, distractibility, social blunders, and names [30]. Each question was scored on a 5-point Likert scale. The total score on the CFQ ranges from 0 to 100; a higher score indicates more-severe cognitive dysfunctioning. The subjective CFQ shows good correlation with more-quantitative mental health tests [29]. An extensive description concerning the use of the CFQ in critically ill patients was recently published [31].

### Statistical analysis

Differences in baseline characteristics between delirious and nondelirious patients were tested by using χ^2 ^tests and the Mann-Whitney *U *or Student *t *tests, depending on its measure and distribution. Biomarkers and CFQ data were successfully log transformed to obtain normally distributed data. To determine the association between biomarkers and delirium in the inflamed and the noninflamed group of patients, univariate logistic regression analysis was performed. To examine the associations between the biomarkers and delirium, a multivariate logistic regression analysis *method backward conditional *was performed, including biomarkers with the 10 best-associated biomarkers in the univariate analysis. Correlations between biomarkers and CFQ outcomes, measured 18 months after ICU discharge, were determined by using Pearson's correlation coefficients.

Because of the exploratory nature of this study, no correction for multiple testing was performed to increase sensitivity. Statistical significance was defined as a *P *value < 0.05.

All data were analyzed by using SPSS version 16.01 (SPSS, Chicago, IL, USA).

## Results

In total, 105 patients were screened for this study, of which five patients were excluded for reasons of doubt concerning the delirium diagnosis or history of cognitive dysfunction of the patients. In three patients, it was not possible to retrieve information about the relatives, and in two patients, some doubt existed concerning the delirium diagnosis (in both cases, the CAM-ICU was negative). In total, 100 patients were included for this study, of whom 50 patients were delirious during the ICU stay, and 50 patients were not delirious during the ICU stay. No statistically significant differences in demographic variables and several clinical covariates related to delirium were observed between groups of 50 delirious and 50 nondelirious patients (Table 1). No difference was noted between both groups regarding the moment of blood withdrawal counted from ICU admission (Table 1). Several pro- and antiinflammatory cytokines, PCT as marker of inflammation, stress response hormone cortisol, as well as several brain-specific proteins differed significantly between delirious and nondelirious patients (Table 2). Of note, all measured levels of biomarkers were well above the lower detection limit. Differences in biomarkers between inflamed and noninflamed delirious patients are illustrated in Additional file 1.

**Table 1:** Demographic variables of delirium and nondelirium ICU patients

	Delirium (*n *= 50)	Nondelirium (*n *= 50)	*P *value
Age in years (95% CI)	72 (38-86)	68 (31-84)	0.10
Gender (male, *n*, %)	27 (46)	26 (40)	0.50
Medical patients (*n*, %)	14 (28)	13 (26)	0.50
Unplanned admissions (*n*, %)	23 (46)	7 (35)	0.34
APACHE-II score (point) (95% CI)	16 (9-25)	15 (6-23)	0.11
Inflamed patients (*n*, %)	26 (52)	20 (40)	0.16
Days on the ICU (median [IQR] before blood drawn	1 [1,2]	1 [1-3]	0.38
Mean arterial blood pressure in mm Hg (95% CI)	64 (47-90)	65 (50-105)	0.37
Use of sedatives (midazolam, propofol) (*n*, %)	28 (56)	16 (32)	0.13
Use of opiates (*n*, %)	45 (90)	42 (84)	0.28
Urea in mmol/L (95% CI)	11 (4-29)	9 (4-23)	0.14
Metabolic acidosis (*n*, %)	15 (30)	14 (28)	0.50

Data are expressed as mean and standard deviation (±), unless other reported.

**Table 2:** Differences between delirious and nondelirious patients

	Delirium (*n *= 50)		Nondelirium (*n *= 50)		Differences *P *value
**Proinflammatory cytokines**					
TNF-α (pg/ml)	11	[7-14]	8	[5-13]	0.02
IL-1*β *(pg/ml)	3	[3-6]	3	[3-7]	0.63
IL-6 (pg/ml)	61	[37-113]	37	[23-81]	0.01
IL-8 (pg/ml)	29	[20-39]	17	[9-26]	< 0.0001
IL-17 (pg/ml)	3	[3-4]	3	[3-4]	0.17
IL-18 (pg/ml)	99	[74-161]	86	[70-120]	0.11
MIF (pg/ml)	418	[300-724]	257	[157-576]	0.02
**Antiinflammatory cytokines**					
IL-1ra (pg/ml)	36	[17-68]	21	[13-33]	0.001
IL-10 (pg/ml)	29	[16-51]	18	[9-39]	0.01
**Chemotactic cytokines**					
MCP-1 (pg/ml)	372	[248-589]	239	[179-325]	< 0.0001
**Defensin**					
HNP (μg/ml)	0.06	[0.03-0.12]	0.06	[0.03-0.10]	0.89
**Markers of inflammation**					
CRP (mg/ml)	56	[35-114]	47	[32-84]	0.11
Procalcitonin (ng/ml)	0.35	[0.17-1.68]	0.14	[0.07-0.36]	< 0.0001
**Stress-response hormone**					
Cortisol (μmol/L)	0.51	[0.31-0.97]	0.35	[0.09-0.62]	0.006
**Brain-specific proteins**					
S100-*β *(pg/ml)	132	[100-294]	135	[90-219]	0.40
Tau (pg/ml)	41	[24-91]	32	[17-56]	0.07
Ratio tau/A*β*_1-42_	1.14	[0.62-2.71]	0.95	[0.47-1.69]	0.10
A*β*_1-42 _(pg/ml)	36	[29-47]	36	[30-41]	0.70
A*β*_1-40 _(pg/ml)	156	[129-225]	146	[113-163]	0.05
Ratio A*β*_1-42/40_	0.23	[0.20-0.27]	0.25	[0.23-0.30]	0.006
A*β*_N-42 _(pg/ml)	31	[23-40]	29	[24-36]	0.65
A*β*_N-40 _(pg/ml)	222	[167-276]	178	[146-220]	0.02
Ratio A*β *_N-42/40_	0.15	[0.12-0.17]	0.17	[0.14-0.19]	0.04
Ratio A*β*_1-42/N-42_	1.26	[1.03-1.34]	1.29	[1.10-1.40]	0.48
Ratio A*β*_1-40/N-40_	0.78	[0.69-0.85]	0.80	[0.73-0.92]	0.28

Data are expressed as median and IQR. Differences were tested with the Mann-Whitney *U *test.

^a^*P *value < 0.05.

A*β*_1-42/40_, amyloid*β*_1-42 _and _1-40_; A*β*_N-42/40_, amyloid*β *truncated_-42 _and _1; _CRP, C-reactive protein; HNP, human neutrophil protein-1; IL, interleukin; MCP, monocyte chemotactic protein 1; MIF, macrophage migration inhibitory factor; NSE, neurospecific enolase; S100-*β*, S100 calcium-binding protein-*β *(S100-*β*);

### Inflamed patients

This group consisted of 26 delirium and 20 nondelirium ICU patients. Several pro- as well as antiinflammatory cytokines, PCT, and cortisol were significantly higher in the delirium group compared with the nondelirium group (Table 3). Levels of brain-specific proteins were comparable between the groups, except for a borderline-significant elevated level of S100-*β *in the delirium group (*P = *0.07). In univariate logistic regression analysis, IL-8, MCP-1, PCT, cortisol, and S100-*β *were significantly (*P <*0.05) associated with delirium. Extended with the biomarkers TNF-α, IL-6, IL-18, IL-1ra, and IL-10, the 10 best biomarkers associated with delirium (all *P *< 0.10) were then entered into a multivariate logistic regression analysis. This multivariate analysis demonstrated a significant association between the proinflammatory cytokine IL-8 (odds ratio, 9.0; 95% CI, 1.8 to 44.0) with the presence of delirium in inflamed patients.

**Table 3:** Differences between delirium and nondelirium patients in inflamed and noninflamed patients

	Inflamed (*n *= 46)					Noninflamed patients (*n *= 54)				
	**Delirium (*n *= 26)**		**Nondelirium (*n *= 20)**		**P value**	**Delirium (*n *= 24)**		**Nondelirium (*n *= 30)**		***P *value**
**Proinflammatory cytokines**										
TNF-α (pg/ml)	13	[10-16]	11	[5-18]	0.17	8	[5-13]	7	[5-11]	0.18
IL-1*β *(pg/ml)	3	[3-6]	4	[3-17]	0.67	3	[3-6]	3	[3-6]	0.69
IL-6 (pg/ml)	73	[38-143]	41	[21-90]	0.09	50	[29-90]	34	[22-64]	0.047^a^
IL-8 (pg/ml)	31	[24-44]	17	[9-26]	< 0.001^a^	20	[12-32]	14	[9-22]	0.001^a^
IL-17 (pg/ml)	4	[3-7]	3	[3-6]	0.22	3	[3-4]	3	[3-3]	0.63
IL-18 (pg/ml)	136	[88-187]	84	[65-132]	0.03^a^	82	[66-141]	88	[72-120]	0.54
MIF (pg/ml)	438	[294-796]	257	[157-576]	0.13	334	[214-561]	249	[179-702]	0.08
**Antiinflammatory cytokines**										
IL-1ra (pg/ml)	48	[27-74]	32	[18-47]	0.04^a^	24	[17-51]	16	[11-25]	0.02^a^
IL-10 (pg/ml)	23	[13-47]	13	[5-35]	0.08	28	[12-44]	22	[9-46]	0.03^a^
**Chemotactic cytokines**										
MCP-1 (pg/ml)	516	[295-822]	251	[199-339]	0.001^a^	268	[192-398]	233	[175-306]	0.15
**Defensin**										
HNP (μg/ml)	0.06	[0.03-0.13]	0.07	[0.03-0.09]	0.60	0.06	[0.04-0.10]	0.04	[0.03-0.10]	0.51
**Markers of inflammation**										
CRP (mg/ml)	84	[56-190]	84	[43-140]	0.40	42	[29-65]	41	[27-64]	0.44
Procalcitonin (ng/ml)	1.0	[0.23-2.0]	0.28	[0.10-0.64]	0.003^a^	0.22	[0.11-0.55]	0.12	[0.06-0.18]	0.01^a^
**Stress-response hormone**										
Cortisol (μmol/L)	0.59	[0.34-0.98]	0.48	[0.18-0.61]	0.06	0.46	[0.23-0.72]	0.30	[0.06-0.66]	0.06
**Brain-specific proteins**										
S100-*β *(pg/ml)	172	[113-409]	134	[88-163]	0.07	128	[87-210]	136	[92-247]	0.60
Tau (pg/ml)	42	[26-131]	43	[24-75]	0.56	40	[21-78]	27	[17-46]	0.08
Ratio tau/A*β*_1-42_	1.03	[0.62-3.45]	1.12	[0.40-2.21]	0.68	1.17	[0.60-2.52]	0.90	[0.48-1.26]	0.07
A*β*_1-42 _(pg/ml)	41	[31-52]	38	[31-42]	0.36	34	[26-43]	36	[30-42]	0.55
A*β*_1-40 _(pg/ml)	158	[132-229]	155	[137-178]	0.55	148	[109-223]	129	[106-158]	0.08
Ratio A*β*_1-42/40_	0.23	[0.20-0.28]	0.24	[0.22-0.26]	0.72	0.22	[0.19-0.26]	0.26	[0.23-0.33]	0.001^a^
A*β*_N-42 _(pg/ml)	31	[26-43]	29	[24-39]	0.57	28	[20-37]	28	[24-35]	0.79
A*β*_N-40 _(pg/ml)	200	[167-283]	184	[147-229]	0.24	225	[168-273]	178	[145-220]	0.04^a^
Ratio A*β *_N-42/40_	0.16	[0.13-0.18]	0.18	[0.12-0.19]	0.47	0.13	[0.10-0.17]	0.16	[0.14-0.20]	0.02^a^
Ratio A*β*_1-42/N-42_	1.28	[1.00-1.39]	1.31	[1.18-1.48]	0.26	1.24	[1.04-1.33]	1.23	[1.05-1.39]	0.90
Ratio A*β*_1-40/N-40_	0.82	[0.74-0.89]	0.89	[0.73-0.96]	0.27	0.72	[0.65-0.84]	0.76	[0.70-0.87]	0.35

Data are expressed as median and IQR. Differences were tested with the Mann-Whitney *U *test.

^a^*P *value < 0.05.

### Noninflamed patients

This group of patients consisted of 24 delirium and 30 nondelirium ICU patients. The proinflammatory cytokines IL-6 and IL8 as well as the antiinflammatory cytokines IL-1ra and IL-10 and level of PCT were significantly higher in delirious patients compared with the nondelirious patients. Furthermore, several amyloid-*β *forms differed significantly, and Tau levels differed borderline significantly between the two groups (Table 3).

Biomarkers that were significantly associated with delirium were IL-8, IL-1ra, IL-10, ratio A*β*_1-42/40_, and ratio A*β*_N-42/40_. Furthermore IL-6, PCT, cortisol, ratio Tau/A*β*_1-42_, A*β*_1-40_, and A*β*_N-40 _were in total the 10 best, with delirium-associated biomarkers (all *P < 0.10) *in univariate logistic regression analysis. Multivariate logistic regression analyses with these biomarkers showed a significant association of ratio A*β-*_42/40 _(OR, 0.03; 95% CI, 0.002 to 0.50) and the antiinflammatory cytokine IL-10 (OR, 2.6; 95% CI, 1.1 to 5.9) with the presence of delirium in noninflamed patients.

### Correlations of biomarkers with long-term subjective cognitive failure

At a median of 18 months after ICU discharge, 10 of the 100 ICU patients had died. Except for a significantly lower level of IL-1*β *in the survivors group, no other differences were found between the survivors and nonsurvivors (data not shown).

In total, 52 (58%) patients of the 90 survivors returned the CFQ; 23 (44%) of these were delirious during their ICU stay. No important differences were noted between nonresponders and responders concerning age (69 ± 8 versus 73 ± 6 years; *P = 0.12*), APACHE-II (16 ± 3 versus 15 ± 4; *P = 0.31*), gender (male, 46%, versus 54%; *P = 0.45*), delirium (46% versus 44%; *P = 0.60*), and inflamed (55% versus 40%; *P = 0.29*).

The A*β_1-40 _*and the A*β_1-42/40 _*ratio correlated with the domain distractibility *(r *= 0.30; *P *= 0.03; and *r *= -0.28; *P *= 0.04, respectively). The *Aβ_N-42/40 _*ratio correlated with the domain memory *(r *= -0.30; *P *= 0.03). *Aβ_N-40 _*correlated with the domain social blunders *(r *= 0.31; *P *= 0.03). A*β_N-40 _*and the A*β_N-42/40 _*ratio correlated significantly with the total CFQ score *(r *= 0.30; *P *= 0.04; and *r *= -0.34; *P *= 0.02, respectively).

We found no correlation between S100-*β*, total human Tau, cortisol, or any of the other measured inflammatory mediators, CFQ, age, APACHE-II score, and mean blood pressure. In addition, we found no correlations between CFQ and age, APACHE-II score, and length of stay in the ICU.

The patient numbers per subgroup were too low and therefore did not allow us to perform correlation analyses between biomarkers and the different subgroups because of lack of statistical power.

## Discussion

This study shows that differences exist in various inflammatory mediators associated with delirium between inflamed and noninflamed patients. After multivariate regression analysis, IL-8 was associated with delirium in inflamed patients, whereas in noninflamed patients, IL-10 and A*β-*_42/40 _were associated with delirium. These differences between inflamed and noninflamed ICU patients in delirium-associated biomarkers suggest that the underlying mechanism governing the development of delirium in inflamed patients differs from that in noninflamed patients. Furthermore, we demonstrated that, in contrast to inflammatory mediators, different forms of amyloid*β *significantly correlate with long-term subjective cognitive problems in ICU patients, illustrating that the underlying mechanism of delirium is relevant for its long-term cognitive consequences.

This is the first study investigating plasma amyloid*β (*A*β) *levels and human Tau in critically ill patients in relation to the presence of delirium. In view of the reported increased incidence of dementia after ICU/hospital admission [16], our findings could provide a possible mechanistic link, because noninflamed delirium is associated with A*β*, but this must be confirmed in a longitudinal study focusing on these biomarkers combined with more-extensive cognitive testing. Furthermore, A*β *is associated with sustained long-term subjective cognitive dysfunction in ICU patients. Studies comparing plasma levels of A*β *between Alzheimer (AD) and non-Alzheimer dementia patients and controls [17,18,32] have yielded conflicting results with respect to levels of different forms of A*β*. Increased levels of A*β_1-42 _*[17] as well as increased levels of A*β_1-40 _*[18] were found in dementia patients [32]. In addition, increased levels of the Tau/A*β_1-42 _*ratio have been found in cerebrospinal fluid (CSF) of patients with cerebral amyloid deposition [33], but this has not yet been investigated in plasma. In the present study, the difference in levels of total Tau and the Tau/A*β_1-42 _*ratio between noninflamed delirious patients and noninflamed nondelirious patients approached statistical significance. It is known that plasma levels of A*β *are age dependent [34]; however, this could not have confounded our results because no differences in age existed between delirious and nondelirious patients in our study. Additionally, the patients investigated in this study were not recognized with a history of cognitive impairment by patients' medical history and information from their relatives, which could explain differential A*β *levels. The lower A*β_1-42/40 _*ratio, probably due to an increase of A*β_1-40 _*at a constant A*β_1-42 _*level, in combination with a significant correlation with long-term cognitive failure on several domains of the CFQ are in accordance with findings that elevated levels of A*β_1-40 _*increase the risk of developing dementia [16,18]. Importantly, this finding of early lower A*β_1-42/40 _*ratio and ratio A*β_1-40/N-40 _*in delirious patients without signs of serious previous cognitive impairment makes it tempting to speculate that this represents the first sign of an imbalance in the A*β *metabolism. To our knowledge, these early findings of imbalance in A*β *metabolism have not been reported. Our findings might therefore shed new light on the important question whether delirium plays a causative role in the development of dementia in later life, or if delirium is the first sign of dementia. Because deposition of A*β *in the brain is generally considered to be a long-term process, and samples in our study were drawn shortly after the onset of delirium, it is more plausible that delirium may be the first sign of an early dementia process. However, a cause-effect relation cannot be determined in a cross-sectional observational study like ours. This hypothesis of early imbalance in A*β *metabolism in delirious patients must be confirmed in future studies.

Previously, it was demonstrated that delirium is associated with elevated levels of IL-6, IL-8, and S100-*β *in non-ICU patients [9,10] and with IL-6 and S100-*β *in ICU patients with sepsis [13]. IL-8 levels were not measured in these sepsis patients. We showed, by using a multivariate logistic regression analysis, that levels of IL-8 in inflamed patients were associated with delirium, but IL-6 was not. A possible reason for this discrepancy might be that we determined biomarkers directly after the first positive delirium screening, whereas it has been shown that the highest levels of IL-6 occur in the later phase of delirium [10].

Several limitations of our study should be addressed. First, we used the CAM-ICU to diagnose delirium in ICU patients instead of the gold standard: the DSM-IV criteria [35]. It is recognized that it is not feasible to use this gold standard in ICU patients, and therefore the CAM-ICU is an accepted alternative to diagnose delirium in the ICU. The CAM-ICU has the highest sensitivity and specificity rate of all delirium-assessment tools [36,37] and is well implemented in the daily practice of our nurses with a high interrater reliability [26]. In addition, to strengthen the delirium diagnosis, all medical and nurse files of the patients were analyzed, and patients were not included when in doubt of the delirium diagnosis.

Second, we did not use a validated cognitive-assessment tool such as the informant questionnaire on cognitive decline short form (IQCODE-sf), which is a surrogate evaluation to determine whether the patient had serious cognitive impairment before ICU admission. Instead of this, we used information from medical records and the next of kin of the patients to identify whether the patient had a history of cognitive impairment. In case of any reference to or sign of cognitive impairment, patients were not included in our study. Furthermore, as a measure of patients' cognitive function 18 months after ICU discharge, we used the validated CFQ, which is a self-evaluated questionnaire to detect cognitive-based failures and not dementia and is also not a specific psychometric test, which may result in more-objective data. Although this can be considered a limitation of our study, our findings are the results of patient's own perception of cognitive functioning and are therefore informative and relevant.

Third, in this study, we measured biomarkers only at one point in time. In a longitudinal biomarker study [9], a difference in cytokine levels before and during delirium was found. In the absence of biomarker data in critically ill patients with delirium, we chose to perform an exploratory study to investigate which biomarkers were most strongly associated with delirium immediately after the onset of delirium. This was an exploratory hypothesis-generating study, of which the results may facilitate hypotheses for future research.

Fourth, potential covariates must be considered as a potential limitation of the study, in contrast to a randomized trial in which possible covariates are likely to be equally divided between the groups. Although baseline patient characteristics were comparable between the delirium and nondelirium groups, unbalanced influence of covariates cannot be ruled out in such an observational study as we performed.

Last, we measured levels of brain biomarkers in peripheral blood and not directly in material derived from the brain or cerebrospinal fluid. It is recognized that levels of A*β*_1-42 _in cerebrospinal fluid of AD patients are decreased [38], but studies on plasma A*β *forms have yielded ambiguous results [18,39-43]. A large prospective study showed that increased plasma levels of A*β*_1-40 _increased the risk for dementia, especially when the concentration of A*β*_1-42 _was increased [18]. This results in a decrease of ratio A*β*_1-42/40 _[40]. A combination of different brain-specific proteins, such as a combination of A*β *with Tau concentrations in CSF, improves discrimination between AD patients and controls [44]. Although it has been recommended to determine these biomarkers in CSF rather than in plasma [45], our results are in accordance with these findings. Interestingly, levels of Tau, ratio Tau/A*β*_1-42_, and A*β*_1-40 _were increased in inflamed delirious and nondelirious patients and in delirious noninflamed patients, but appear to be lower in nondelirious noninflamed patients. It can be argued that a blood-barrier change during systemic inflammation may play a role. This may also suggest that determining neuronal biomarkers in plasma can be used instead of only CSF samples. Obviously, CSF samples are not routinely obtained in our ICU patients. To our knowledge, a study investigating the correlation between CSF and plasma levels of A*β *has yet to be performed.

## Conclusions

In inflamed patients, the proinflammatory cytokine IL-8 was independently associated with delirium, whereas in noninflamed patients, the ratio A*β*_*1-*42/40 _and IL-10 were independently associated with delirium, as determined by multivariate regression analyses. This suggests that the underlying mechanism governing the development of delirium in inflamed patients differs from that in noninflamed patients. These findings illustrate the relevance of distinguishing between inflamed and noninflamed when investigating biomarkers in delirious patients. Finally, elevated levels of amyloid-β correlated with long-term cognitive impairment. These findings are in line with the notion that delirium in noninflamed ICU patients may represent the first sign of a (subclinical) dementia process. Future research into the relation of delirium, amyloid forms, and long-term cognitive function should include more-extensive tests of cognitive function.

## Key messages

• Two different pathways exist in delirious inflamed and noninflamed ICU patients

• Delirium in inflamed patients is independently associated with the proinflammatory cytokine IL-8, and in noninflamed patients, delirium is independently associated with the antiinflammatory cytokine IL-10 and the ratio amyloid*β-*_42/40_.

• These increased amyloid-*β *concentrations, but not increased cytokine concentrations correlate with long-term cognitive failure.

## Abbreviations

A*β*_1-42/40_: amyloid*β*_1-42 _and _1-40_; A*β*_N-42/40_: amyloid*β *truncated_-42 _and _1-40_; AD: Alzheimer disease; APACHE-II: acute physiology and chronic health evaluation-II; BSP: brain-specific proteins; CAM-ICU: confusion-assessment method, intensive care unit; CFQ: cognitive failure questionnaire; CRP: C-reactive protein; CSF: cerebrospinal fluid; DSM-IV: Diagnostic and Statistical Manual of Mental Disorders-IV; HNP: human neutrophil protein-1; IQCODE-sf: Informant Questionnaire on Cognitive Decline Short Form; IL: interleukin; MCP: monocytes chemotactic protein 1; MIF: macrophage migration inhibitory factor; RASS: Richmond Agitation Sedation Score; S100-*β*: S100 calcium-binding protein-*β*; SIRS: systemic inflammatory response syndrome.

## Conflicting interests

The authors declare that they have no competing interests.

## Authors' contributions

MvdB carried out the study, collected all data and blood, performed the statistical analysis, and drafted the manuscript. MK measured plasma biomarkers and drafted the manuscript. PP and LS supervised the conduct of the study and writing of the article. KQ measured plasma biomarkers and corrected the manuscript. JvdH and TvA co-supervised and corrected the manuscript.

All authors read and approved the final manuscript.

## Supplementary Material

Additional file 1 **Differences between inflamed and noninflamed delirium patients**. Differences in measured levels of cytokines, stress-response hormone, and several brain-specific proteins between inflamed and noninflamed delirious patients. Click here for file

## References

[B1] Ely EW Gautam S Margolin R Francis J May L Speroff T Truman B Dittus R Bernard R Inouye SK The impact of delirium in the intensive care unit on hospital length of stay Intensive Care Med 2001 27 1892 900 10.1007/s00134-001-1132-2 11797025 PMC7095464

[B2] Ely EW Shintani A Truman B Speroff T Gordon SM Harrell FE Jr Inouye SK Bernard GR Dittus R Delirium as a predictor of mortality in mechanically ventilated patients in the intensive care unit JAMA 2004 292 168 9 15082703 10.1001/jama.291.14.1753

[B3] Girard TD Jackson JC Pandharipande PP Pun BT Thompson JL Shintani AK Gordon SM Canonico AE Dittus RS Bernard GR Ely EW Delirium as a predictor of long-term cognitive impairment in survivors of critical illness Crit Care Med 2010 38 1513 20 10.1097/CCM.0b013e3181e47be1 20473145 PMC3638813

[B4] van den Boogaard M Schoonhoven L Evers AW van der Hoeven JG van AT Pickkers P Delirium in critically ill patients: impact on long-term health-related quality of life and cognitive functioning Crit Care Med 2012 40 112 8 10.1097/CCM.0b013e31822e9fc9 21926597

[B5] Flacker JM Lipsitz LA Neural mechanisms of delirium: current hypotheses and evolving concepts J Gerontol A Biol Sci Med Sci 1999 54 B239 46 10.1093/gerona/54.6.B239 10411009

[B6] Maclullich AM Ferguson KJ Miller T de Rooij SE Cunningham C Unravelling the pathophysiology of delirium: a focus on the role of aberrant stress responses J Psychosom Res 2008 65 229 38 10.1016/j.jpsychores.2008.05.019 18707945 PMC4311661

[B7] Marcantonio ER Rudolph JL Culley D Crosby G Alsop D Inouye SK Serum biomarkers for delirium J Gerontol A Biol Sci Med Sci 2006 61 1281 6 10.1093/gerona/61.12.1281 17234821

[B8] de Rooij SE Van Munster BC Korevaar J Levi M Cytokines and acute phase response in delirium J Psychosom Res 2007 62 521 5 10.1016/j.jpsychores.2006.11.013 17467406

[B9] van Munster BC Bisschop PH Zwinderman AH Korevaar JC Endert E Wiersinga WJ Oosten HE Goslings JC de Rooij SE Cortisol, interleukins and S100B in delirium in the elderly Brain Cogn 2010 in press 10.1016/j.bandc.2010.05.010 20580479

[B10] Van Munster BC Korevaar JC Zwinderman AH Levi M Wiersinga WJ de Rooij SE Time-course of cytokines during delirium in elderly patients with hip fractures J Am Geriatr Soc 2008 56 1704 9 10.1111/j.1532-5415.2008.01851.x 18691278

[B11] Van Munster BC Korevaar JC Korse CM Bonfrer JM Zwinderman AH de Rooij SE Serum S100B in elderly patients with and without delirium Int J Geriatr Psychiatry 2009 25 234 9 19575407 10.1002/gps.2326

[B12] Iacobone E Bailly-Salin J Polito A Friedman D Stevens RD Sharshar T Sepsis-associated encephalopathy and its differential diagnosis Crit Care Med 2009 37 S331 6 20046118 10.1097/CCM.0b013e3181b6ed58

[B13] Pfister D Siegemund M ll-Kuster S Smielewski P Ruegg S Strebel SP Marsch SC Pargger H Steiner LA Cerebral perfusion in sepsis-associated delirium Crit Care 2008 12 R63 10.1186/cc6891 18457586 PMC2481444

[B14] Gunther ML Jackson JC Wesley EE Loss of IQ in the ICU brain injury without the insult Med Hypotheses 2007 69 1179 82 10.1016/j.mehy.2007.03.039 17555884

[B15] Alexander JJ Jacob A Cunningham P Hensley L Quigg RJ TNF is a key mediator of septic encephalopathy acting through its receptor, TNF receptor-1 Neurochem Int 2008 52 447 56 10.1016/j.neuint.2007.08.006 17884256 PMC3191465

[B16] Witlox J Eurelings LS de Jonghe JF Kalisvaart KJ Eikelenboom P van Gool WA Delirium in elderly patients and the risk of postdischarge mortality, institutionalization, and dementia: a meta-analysis JAMA 2010 304 443 51 10.1001/jama.2010.1013 20664045

[B17] Pesaresi M Lovati C Bertora P Mailland E Galimberti D Scarpini E Quadri P Forloni G Mariani C Plasma levels of beta-amyloid (1-42) in Alzheimer's disease and mild cognitive impairment Neurobiol Aging 2006 27 904 5 10.1016/j.neurobiolaging.2006.03.004 16638622

[B18] van Oijen M Hofman A Soares HD Koudstaal PJ Breteler MM Plasma Abeta(1-40) and Abeta(1-42) and the risk of dementia: a prospective case-cohort study Lancet Neurol 2006 5 655 60 10.1016/S1474-4422(06)70501-4 16857570

[B19] Lesur O Roussy JF Chagnon F Gallo-Payet N Dumaine R Sarret P Chraibi A Chouinard L Hogue B Proven infection-related sepsis induces a differential stress response early after ICU admission Crit Care 2010 14 R131 10.1186/cc9102 20615266 PMC2945098

[B20] Brenner T Hofer S Rosenhagen C Steppan J Lichtenstern C Weitz J Bruckner T Lukic IK Martin E Bierhaus A Hoffmann U Weigand MA Macrophage migration inhibitory factor (MIF) and manganese superoxide dismutase (MnSOD) as early predictors for survival in patients with severe sepsis or septic shock J Surg Res 2010 164 e163 71 10.1016/j.jss.2010.05.004 20863520

[B21] Quinn K Henriques M Parker T Slutsky AS Zhang H Human neutrophil peptides: a novel potential mediator of inflammatory cardiovascular diseases Am J Physiol Heart Circ Physiol 2008 295 H1817 24 10.1152/ajpheart.00472.2008 18805897 PMC4896811

[B22] Aldemir M Ozen S Kara IH Sir A Bac B Predisposing factors for delirium in the surgical intensive care unit Crit Care 2001 5 265 70 10.1186/cc1044 11737901 PMC83853

[B23] Ely EW Inouye SK Bernard GR Gordon S Francis J May L Truman B Speroff T Gautam S Margolin R Hart RP Dittus R Delirium in mechanically ventilated patients: validity and reliability of the confusion assessment method for the intensive care unit (CAM-ICU) JAMA 2001 286 2703 10 10.1001/jama.286.21.2703 11730446

[B24] Ely EW Margolin R Francis J May L Truman B Dittus R Speroff T Gautam S Bernard GR Inouye SK Evaluation of delirium in critically ill patients: validation of the Confusion Assessment Method for the Intensive Care Unit (CAM-ICU) Crit Care Med 2001 29 1370 9 10.1097/00003246-200107000-00012 11445689

[B25] American College of Chest Physicians/Society of Critical Care Medicine Consensus Conference Definitions for sepsis and organ failure and guidelines for the use of innovative therapies in sepsis Crit Care Med 1992 20 864 74 10.1097/00003246-199206000-00025 1597042

[B26] van den Boogaard M Pickkers P van der Hoeven H Roodbol G van Achterberg T Schoonhoven L Implementation of a delirium assessment tool in the ICU can influence haloperidol use Crit Care 2009 13 R131 10.1186/cc7991 19664260 PMC2750188

[B27] Lachno DR Vanderstichele H De GG Kostanjevecki V De MG Siemers ER Willey MB Bourdage JS Konrad RJ Dean RA The influence of matrix type, diurnal rhythm and sample collection and processing on the measurement of plasma beta-amyloid isoforms using the INNO-BIA plasma Abeta forms multiplex assay J Nutr Health Aging 2009 13 220 5 10.1007/s12603-009-0062-5 19262957 PMC12876343

[B28] Merckelbach H Muris P Nijman H de Jong PJ Self-reported cognitive failures and neurotic symptomatology Personal Individ Differ 1996 20 715 24 10.1016/0191-8869(96)00024-4

[B29] Broadbent DE Cooper PF FitzGerald P Parkes KR The Cognitive Failures Questionnaire (CFQ) and its correlates Br J Clin Psychol 1982 21 Pt 1 1 16 7126941 10.1111/j.2044-8260.1982.tb01421.x

[B30] Wallace JC Kass SJ Stanny CJ The Cognitive Failures Questionnaire revisited: dimensions and correlates J Gen Psychol 2002 129 238 56 10.1080/00221300209602098 12224809

[B31] van den Boogaard M Schoonhoven L Evers AW van der Hoeven JG van AT Pickkers P Delirium in critically ill patients: impact on long-term health-related quality of life and cognitive functioning Crit Care Med 2012 40 112 8 10.1097/CCM.0b013e31822e9fc9 21926597

[B32] Le Bastard N Leurs J Blomme W De Deyn PP Engelborghs S Plasma amyloid-beta forms in Alzheimer's disease and non-Alzheimer's disease patients J Alzheimers Dis 2010 21 291 301 20421698 10.3233/JAD-2010-091501

[B33] Fagan AM Mintun MA Shah AR Aldea P Roe CM Mach RH Marcus D Morris JC Holtzman DM Cerebrospinal fluid tau and ptau(181) increase with cortical amyloid deposition in cognitively normal individuals: implications for future clinical trials of Alzheimer's disease EMBO Mol Med 2009 1 371 80 10.1002/emmm.200900048 20049742 PMC2806678

[B34] Takeda S Sato N Rakugi H Morishita R Plasma beta-amyloid as potential biomarker of Alzheimer disease: possibility of diagnostic tool for Alzheimer disease Mol Biosyst 2010 6 1760 6 10.1039/c003148h 20567751

[B35] American Psychiatric Association Diagnostic and Statistical Manual of Mental Disorders (DSM-IV) 1994 4 Washington, DC: American Psychiatric Association

[B36] Devlin JW Fong JJ Fraser GL Riker RR Delirium assessment in the critically ill Intensive Care Med 2007 33 929 40 10.1007/s00134-007-0603-5 17401550

[B37] van den Boogaard M Pickkers P Schoonhoven L Assessment of delirium in ICU patients; a literature review Netherlands J Crit Care 2010 14 10 15

[B38] Hampel H Teipel SJ Fuchsberger T Andreasen N Wiltfang J Otto M Shen Y Dodel R Du Y Farlow M Moller HJ Blennow K Buerger K Value of CSF beta-amyloid1-42 and tau as predictors of Alzheimer's disease in patients with mild cognitive impairment Mol Psychiatry 2004 9 705 10 14699432 10.1038/sj.mp.4001473

[B39] Blennow K Hampel H Weiner M Zetterberg H Cerebrospinal fluid and plasma biomarkers in Alzheimer disease Nat Rev Neurol 2010 6 131 44 10.1038/nrneurol.2010.4 20157306

[B40] Graff-Radford NR Crook JE Lucas J Boeve BF Knopman DS Ivnik RJ Smith GE Younkin LH Petersen RC Younkin SG Association of low plasma Abeta42/Abeta40 ratios with increased imminent risk for mild cognitive impairment and Alzheimer disease Arch Neurol 2007 64 354 62 10.1001/archneur.64.3.354 17353377

[B41] Mayeux R Tang MX Jacobs DM Manly J Bell K Merchant C Small SA Stern Y Wisniewski HM Mehta PD Plasma amyloid beta-peptide 1-42 and incipient Alzheimer's disease Ann Neurol 1999 46 412 6 10.1002/1531-8249(199909)46:3<412::AID-ANA19>3.0.CO;2-A 10482274

[B42] Pomara N Shao B Wisniewski T Mehta PD Decreases in plasma A beta 1-40 levels with aging in non-demented elderly with ApoE-epsilon 4 allele Neurochem Res 1998 23 1563 6 10.1023/A:1020936222286 9821162

[B43] Pomara N Willoughby LM Sidtis JJ Mehta PD Selective reductions in plasma Abeta 1-42 in healthy elderly subjects during longitudinal follow-up: a preliminary report Am J Geriatr Psychiatry 2005 13 914 7 16223971 10.1176/appi.ajgp.13.10.914

[B44] Lewczuk P Esselmann H Otto M Maler JM Henkel AW Henkel MK Eikenberg O Antz C Krause WR Reulbach U Kornhuber J Wiltfang J Neurochemical diagnosis of Alzheimer's dementia by CSF Abeta42, Abeta42/Abeta40 ratio and total tau Neurobiol Aging 2004 25 273 81 10.1016/S0197-4580(03)00086-1 15123331

[B45] Zimmermann R Kornhuber J Lewczuk P [The future of biomarkers in dementia diagnostics.] Nervenarzt 2011 82 1385 86 10.1007/s00115-011-3348-x 21922304

