# Supplementary material for: Biomarkers associated with delirium in critically ill patients and their relation with long-term subjective cognitive dysfunction; indications for different pathways governing delirium in inflamed and noninflamed patients
Source: Crit Care. 2011 Dec 29;15(6):R297. doi: 10.1186/cc10598 (PMC3388649; doi:10.1186/cc10598)
Supplement: Additional file 1 — Differences between inflamed and noninflamed delirium patients. Differences in measured levels of cytokines, stress-response hormone, and several brain-specific proteins between inflamed and noninflamed delirious patients. [file cc10598-S1.DOC]

**Additional file 1. Differences between inflamed and non-inflamed delirium patients**

|  | ***Delirium patients*** | | | | | | |  |
| --- | --- | --- | --- | --- | --- | --- | --- | --- |
|  | ***Inflamed (n=26)*** | | ***Non- inflamed (n=24)*** | | | | ***P-value*** | |
| ***Proinflammatory cytokines*** | | |  |  | |  | |  |
| TNF-α (pg/mL) | 13 | [10-16] | 8 | [5-13] | | *0.03** | |  |
| IL-1*β* (pg/mL) | 3 | [3-6] | 3 | [3-6] | | *0.61* | |  |
| IL-6 (pg/mL) | 73 | [38–143] | 50 | [29–90] | | *0.58* | |  |
| IL-8 (pg/mL) | 31 | [24–44] | 20 | [12–32] | | *0.04** | |  |
| IL-17 (pg/mL) | 4 | [3-7] | 3 | [3-4] | | *0.15* | |  |
| IL-18 (pg/mL) | 136 | [88–187] | 82 | [66-141] | | *0.004** | |  |
| MIF (pg/mL) | 438 | [294–796] | 334 | [214–561] | | *0.66* | |  |
| ***Anti-inflammatory cytokines*** | | |  |  | |  | |  |
| IL-1ra (pg/mL) | 48 | [27–74] | 24 | [17–51] | | *0.02** | |  |
| IL-10 (pg/mL) | 23 | [13–47] | 28 | [12–44] | | *0.15* | |  |
| ***Chemotactic cytokines*** |  |  |  |  | |  | |  |
| MCP-1 (pg/mL) | 516 | [295–822] | 268 | [192–398] | | *0.01** | |  |
| ***Defensin*** |  |  |  | |  |  | |  |
| HNP (µg/mL) | 0.06 | [0.03–0.13] | 0.06 | | [0.04–0.10] | *0.72* | |  |
| ***Markers of inflammation*** | | |  |  | |  | |  |
| CRP (mg/ml) | 84 | [56-190] | 42 | [29-65] | | *0.002** | |  |
| Procalcitonine (ng/mL) | 1.0 | [0.23-2.0] | 0.22 | [0.11-0.55] | | *<0.0001** | |  |
| ***Stress response hormone*** | | |  |  | |  | |  |
| Cortisol (µmol/L) | 0.59 | [0.34–0.98] | 0.46 | [0.23–0.72] | | *0.44* | |  |
| ***Brain Specific Proteins*** |  |  |  |  | |  | |  |
| S100-*β* (pg/ml) | 172 | [113–409] | 128 | [87–210] | | *0.09* | |  |
| Tau (pg/ml) | 42 | [26–131] | 40 | [21-78] | | *0.35* | |  |
| Ratio Tau/A*β*_1-42_ | 1.03 | [0.62-3.45] | 1.17 | [0.60-2.52] | | *0.84* | |  |
| A*β*_1-42_ (pg/ml) | 41 | [31-52] | 34 | [26–43] | | *0.07* | |  |
| A*β*_1-40_ (pg/ml) | 158 | [132–229] | 148 | [109–223] | | *0.45* | |  |
| Ratio A*β*_1-42/40_ | 0.23 | [0.20-0.28] | 0.22 | [0.19–0.26] | | *0.31* | |  |
| A*β*_N-42_ (pg/ml) | 31 | [26-43] | 28 | [20-37] | | *0.23* | |  |
| A*β*_N-40_ (pg/ml) | 200 | [167-283] | 225 | [168-273] | | *0.94* | |  |
| Ratio A*β* _N-42/40_ | 0.16 | [0.13-0.18] | 0.13 | [0.10-0.17] | | *0.04** | |  |
| Ratio A*β*_1-42/N-42_ | 1.28 | [1.00-1.39] | 1.24 | [1.04-1.33] | | *0.64* | |  |
| Ratio A*β*_1-40/N-40_ | 0.82 | [0.74-0.89] | 0.72 | [0.65-0.84] | | *0.03** | |  |

Data are expressed as median and IQR. Differences were tested with Mann-Whitney *U* test

* p-value <0.05

CRP = C-reactive protein

IL = Interleukin

HNP = human neutrophil protein-1

MCP = monocyte chemotactic protein 1

MIF = macrophage migration inhibitory factor

S100-*β* = S100 calcium binding protein-*β* (S100-*β*)

NSE = neurospecific enolase

A*β*_1-42/40_ = amyloid*β*_1-42_ and _1-40_

A*β*_N-42/40_ = amyloid*β* truncated_-42_ and _1-40_
